# Association of the ISAR Score with Clinical and Operational Outcomes in Older Emergency Department Patients

**DOI:** 10.3390/jcm15218329

**Published:** 2026-10-26

**Authors:** Sebastian Breden, Marius Czaja, Lisa Schmid, Nicolas Bubendorfer, Michael Dommasch

**Affiliations:** 1 Department of Orthopedics and Sports Orthopedics, TUM Klinikum, Technical University of Munich, 81675 Munich, Germany; sebastian.breden@mri.tum.de; 2 Department of Emergency Medicine, TUM Klinikum, Technical University of Munich, 81675 Munich, Germany

**Keywords:** Identification of Seniors at Risk, ISAR, geriatric risk screening, emergency department, hospital admission, emergency department length of stay

## Abstract

**Background/Objectives:** The Identification of Seniors at Risk (ISAR) score is widely used to identify older emergency department (ED) patients at increased risk of adverse outcomes. However, its association with immediate disposition and operational outcomes remains incompletely characterized. This study evaluated the association of ISAR scores with hospital admission and emergency department length of stay (EDLOS). **Methods:** This retrospective observational cohort study included ED presentations of patients aged ≥65 years at a tertiary university emergency department in 2024. Of 12,105 eligible presentations, 10,090 (83.4%) had a documented ISAR score and constituted the descriptive cohort; 9401 presentations with classifiable admission status were included in the primary admission analysis. Multivariable logistic regression adjusted for age, sex, presenting complaint, triage category, treating specialty, and referral source. The incremental contribution of ISAR beyond routinely available clinical variables was assessed by nested model comparison and receiver operating characteristic analysis. Prolonged EDLOS was defined as ≥6 h. **Results:** Higher ISAR scores were independently associated with hospital admission and prolonged EDLOS. In the continuous-score admission model, each one-point increase in ISAR was associated with higher odds of admission (OR 1.155, 95% CI 1.121–1.191; *p* < 0.001), although categorical sensitivity analysis demonstrated a nonlinear association with a plateau at higher scores. ISAR alone showed modest discrimination for admission (AUC 0.589, 95% CI 0.577–0.600). Addition of ISAR to the clinical model increased the AUC from 0.696 to 0.705 (ΔAUC = 0.009, 95% CI 0.005–0.012; *p* < 0.001). Median EDLOS increased from 326 min (IQR 215–494) at ISAR 0 to 596 min (IQR 324–1312) at ISAR 6. After adjustment including hospital admission status, each one-point increase in ISAR was associated with higher odds of prolonged EDLOS (OR 1.178, 95% CI 1.143–1.214; *p* < 0.001). **Conclusions:** Higher ISAR scores were associated with both hospital admission and prolonged EDLOS. ISAR provided statistically significant but modest incremental prognostic information beyond routinely available clinical variables and should be regarded as a geriatric risk-screening tool rather than a stand-alone disposition instrument. Prospective studies are needed to determine whether ISAR-guided clinical pathways improve patient or operational outcomes.

## 1. Introduction

Population aging represents one of the most significant challenges facing the healthcare system [1,2]. Across Europe and other industrialized countries, the proportion of older adults continues to increase steadily, resulting in growing demands on emergency and acute care services [1]. Older patients account for a disproportionately large share of emergency department (ED) presentations and frequently require extensive diagnostic workup, multidisciplinary assessment, and hospital admission [2]. These increasing demands occur in the broader context of emergency department crowding, prolonged waiting times, and rising healthcare resource utilization [2,3,4].

Compared with younger adults, older ED patients present with greater clinical complexity. Multimorbidity, polypharmacy, cognitive impairment, sensory deficits, reduced functional reserve, and social vulnerability frequently coexist and complicate both diagnostic and therapeutic decision-making [1,2,5]. Furthermore, older patients are more likely to experience adverse outcomes following emergency department presentation, including hospitalization, prolonged inpatient treatment, functional decline, institutionalization, recurrent emergency department visits, and mortality [2,6]. The growing importance of geriatric emergency medicine has therefore prompted efforts to identify high-risk patients early during emergency department assessment [1].

Traditionally, chronological age has been used as a marker of risk in older patients. However, increasing evidence suggests that age alone is insufficient to explain the substantial heterogeneity observed among older adults [5,7]. Individuals of the same chronological age may differ considerably with respect to physiological reserve, functional capacity, cognitive performance, and vulnerability to stressors. Consequently, the concept of frailty has emerged as a more meaningful indicator of biological age and clinical risk [5,7].

Frailty is generally defined as a state of decreased physiological reserve resulting in increased susceptibility to adverse outcomes following relatively minor stressors [5,8,9]. Numerous studies have demonstrated associations between frailty and mortality, hospitalization, postoperative complications, institutionalization, healthcare utilization, and functional decline [7,10,11,12]. In emergency medicine, frailty has become increasingly recognized as an important determinant of outcomes and resource consumption. Frail patients often require more extensive diagnostic evaluation, longer observation periods, multidisciplinary consultation, and more complex discharge planning than non-frail individuals [1,2,13,14].

Given the time-sensitive nature of emergency care, several screening instruments have been developed to rapidly identify older patients at increased risk of adverse outcomes in the emergency department setting. Among these, the Identification of Seniors at Risk (ISAR) score is one of the most widely used and extensively investigated tools [15,16]. Originally developed by McCusker and colleagues in 1999, the ISAR questionnaire consists of six dichotomous items addressing pre-existing functional dependency, increased need for assistance since the acute illness or injury, recent hospitalization, visual impairment, memory problems, and polypharmacy [15,17]. The resulting score ranges from 0 to 6 points, with higher values indicating increasing vulnerability and risk of adverse outcomes.

Over the past two decades, numerous studies have evaluated the predictive performance of the ISAR score in older patient populations. Higher ISAR scores have been associated with functional decline, recurrent emergency department visits, hospital readmission, institutionalization, and mortality [16,17,18,19]. Consequently, the ISAR has been incorporated into routine screening pathways in many emergency departments and has become one of the most frequently used geriatric screening instruments worldwide. Nevertheless, reported predictive performance has varied considerably across studies, and the optimal role of ISAR screening in routine emergency care remains a matter of ongoing debate [14,15,20,21].

An important limitation of the existing literature is that many studies have focused primarily on long-term outcomes such as mortality, functional decline, or healthcare utilization after discharge [16,17,18,19]. In contrast, comparatively little attention has been paid to immediate operational outcomes that are highly relevant to emergency department workflow and resource allocation. Hospital admission is frequently reported as a secondary endpoint, yet data regarding the relationship between ISAR scores and admission decisions remain heterogeneous [19,21,22,23]. Even less is known about the association between ISAR screening results and emergency department length of stay (EDLOS), despite EDLOS being increasingly recognized as a key quality indicator reflecting patient flow, crowding, resource utilization, and operational efficiency [24].

Moreover, many previously published investigations were limited by selective screening strategies, incomplete screening coverage, convenience sampling, or inclusion of highly selected patient populations [14,15,19,20,21]. Such approaches may introduce selection bias and reduce generalizability. In contrast, routine integration of ISAR screening into the triage process provides an opportunity to evaluate the instrument in a large real-world emergency department population.

Therefore, the aim of the present study was to investigate the association between ISAR score and hospital admission in a large cohort of consecutive patients aged 65 years and older presenting to a tertiary care university emergency department. In addition, we sought to evaluate the relationship between ISAR score and emergency department length of stay, including prolonged EDLOS as a secondary outcome. We hypothesized that increasing ISAR scores would be independently associated with both a higher probability of hospital admission and prolonged emergency department stay.

## 2. Methods

### 2.1. Study Design and Setting

This retrospective observational cohort study was performed in the Emergency Department of a large academic tertiary care center with unrestricted access for self-referred and referred patients. This emergency department serves as a regional referral center and provides emergency care across multiple medical and surgical specialties.

The main aim of the study was to evaluate the association between the Identification of Seniors at Risk (ISAR) score and subsequent hospital admission among older patients presenting to the emergency department.

### 2.2. Ethical Approval

The study was approved by the local institutional review board (Ethics Committee of the Technical University of Munich; reference number 2026-363-S-CT; 8 July 2026). Due to the retrospective nature of the study and the use of routinely collected clinical data, the requirement for individual informed consent was waived.

### 2.3. Study Population

For this study, all cases treated in 2024 by the interdisciplinary Emergency Department of the University Hospital of the Technical University Munich were screened. All patients aged 65 years or older presenting to the emergency department during the study period were eligible for inclusion.

According to the institutional standard operating procedure, ISAR screening is intended for all patients aged 65 years or older as part of the triage process. ED presentations with a documented ISAR score constituted the descriptive cohort. For the primary admission analysis, presentations were additionally required to have a disposition that could be classified as hospital admission or non-admission according to the definitions below.

### 2.4. ISAR Screening

The Identification of Seniors at Risk (ISAR) is a validated screening instrument designed to identify older emergency department patients at increased risk of adverse health outcomes [17]. The ISAR questionnaire consists of six dichotomous items addressing pre-existing functional dependency, increased need for assistance since the acute illness or injury, recent hospitalization, visual impairment, memory problems, and polypharmacy.

At the study institution, ISAR assessment was routinely performed by trained triage nurses during the initial emergency department evaluation and documented electronically as part of the routine triage process. The resulting ISAR score was available to treating physicians during subsequent ED care.

For descriptive analyses, ED presentations were stratified according to their original ISAR score (0–6 points). Additionally, previously established screening thresholds of ISAR ≥ 2 and ISAR ≥ 3 points were evaluated.

### 2.5. Data Collection

Clinical and demographic data were extracted retrospectively from the hospital information system and the electronic emergency department database.

The following variables were collected:

- Age at presentation;
- Sex;
- ISAR score;
- Presenting complaint;
- Triage category (Manchester Triage System);
- Referring source;
- Treating specialty;
- Emergency department length of stay (EDLOS).

Presenting complaints were categorized according to the institutional emergency department classification system. Complaint categories accounting for at least 5% of all emergency department presentations were analyzed separately, whereas less frequent complaint categories were combined into a single category (“Other”) to ensure adequate group sizes for statistical analyses.

Referral source was grouped into three categories: self-presentation, medical referral, and emergency medical services (EMS). Self-presentation included patients presenting independently as well as patients arriving by non-emergency medical transport without a formal medical referral. Medical referral comprised referrals by general practitioners as well as internal and interhospital transfers. EMS included ground ambulance services, physician-staffed emergency medical services, and helicopter emergency medical services.

The unit of analysis was the individual emergency department presentation. The pseudonymized study dataset contained encounter-specific identifiers but no longitudinal patient identifier allowing repeated presentations by the same individual to be linked.

### 2.6. Outcome Definition

The primary outcome was hospital admission following emergency department presentation.

Patients discharged without inpatient admission, including those leaving against medical advice or before physician assessment, were classified as non-admitted. Presentations for which hospital admission could not be meaningfully classified within the study framework—comprising referral for external further treatment, direct transfer to another outpatient department within the hospital, observation cases, and patients who died in the ED—were excluded from the primary admission analysis.

Emergency department length of stay (EDLOS) was defined as the time from emergency department registration to discharge from the emergency department or transfer to an inpatient ward. EDLOS was analyzed as a secondary outcome both as a continuous variable and as a dichotomous variable. For the dichotomous analysis, prolonged EDLOS was defined as an emergency department stay of ≥6 h. This threshold was selected as an operationally meaningful cutoff and has previously been used to define prolonged ED length of stay in emergency care research [25,26].

### 2.7. Statistical Analysis

Continuous variables were summarized as mean values with standard deviations or medians with interquartile ranges, depending on data distribution. Categorical variables were reported as absolute frequencies and percentages.

To assess potential selection associated with missing ISAR documentation, ED presentations with and without a documented ISAR score were compared with respect to age, sex, and triage category. Age was compared using the Mann–Whitney U test, whereas sex and triage category were compared using Pearson’s chi-square test. Cramer’s V was calculated as a measure of effect size for categorical comparisons.

Comparisons between admitted and non-admitted ED presentations were performed using the Mann–Whitney U test for continuous variables and Pearson’s chi-square test for categorical variables.

To assess the association between ISAR score and hospital admission, binary logistic regression analyses were performed. First, a univariable logistic regression model including ISAR score as a continuous variable was calculated. Subsequently, a multivariable logistic regression model was constructed adjusting for age, sex, presenting complaint, triage category, treating specialty, and referral source.

To assess the incremental contribution of the ISAR score beyond routinely available clinical variables, a nested model comparison was performed. A clinical model including age, sex, presenting complaint, triage category, treating specialty, and referral source was first fitted without ISAR, followed by addition of the ISAR score. Changes in model fit were assessed using the likelihood-ratio chi-square test and Nagelkerke’s R^2^, and discrimination was compared using paired receiver operating characteristic curves.

Results are reported as odds ratios (ORs) with corresponding 95% confidence intervals (CIs). Model calibration was assessed using the Hosmer–Lemeshow goodness-of-fit test. Model discrimination was evaluated using receiver operating characteristic (ROC) analysis and calculation of the area under the curve (AUC).

EDLOS was summarized using medians and interquartile ranges due to its right-skewed distribution. Differences in EDLOS across ISAR categories were assessed using the Kruskal–Wallis test, followed by Bonferroni-adjusted pairwise comparisons where appropriate.

Differences in EDLOS between dichotomized ISAR groups were analyzed using the Mann–Whitney U test for the thresholds ISAR ≥2 and ISAR ≥3.

For categorical analyses, prolonged EDLOS was defined as an emergency department stay of ≥6 h. Associations between prolonged EDLOS and dichotomized ISAR thresholds were evaluated using Pearson’s chi-square test.

To identify independent predictors of prolonged EDLOS, binary logistic regression was performed with prolonged EDLOS as the dependent variable. The multivariable model included ISAR score, age, sex, presenting complaint, triage category, treating specialty, and referral source as covariates. Results are reported as odds ratios with 95% confidence intervals. Both EDLOS regression models were restricted to ED presentations with known admission status to ensure an identical analytic sample for model comparison. In a secondary model, hospital admission status was added as a concurrent disposition-related variable to examine whether the association between ISAR and prolonged EDLOS persisted after accounting for processes related to inpatient disposition. Admission status was not considered a baseline confounder, as it arises during the same care pathway as EDLOS.

To assess the assumption of a linear relationship between ISAR score and the log-odds of the outcomes, sensitivity analyses were performed in which ISAR was entered as a categorical variable (0–6), with ISAR 0 as the reference category. Model fit was compared with the corresponding models treating ISAR as a continuous predictor using likelihood-ratio tests.

Statistical significance was defined as a two-sided *p*-value < 0.05. All analyses were performed using IBM SPSS Statistics version 29.0.2.0 (IBM Corp., Armonk, NY, USA).

## 3. Results

### 3.1. Study Population and Patient Characteristics

During the study period, 41,095 ED presentations were recorded, of which 12,105 (29.5%) involved patients aged 65 years or older. A documented ISAR score was available for 10,090 presentations (83.4%), which constituted the descriptive study cohort; 2015 presentations (16.6%) without a documented ISAR score were excluded.

**Figure 1:**
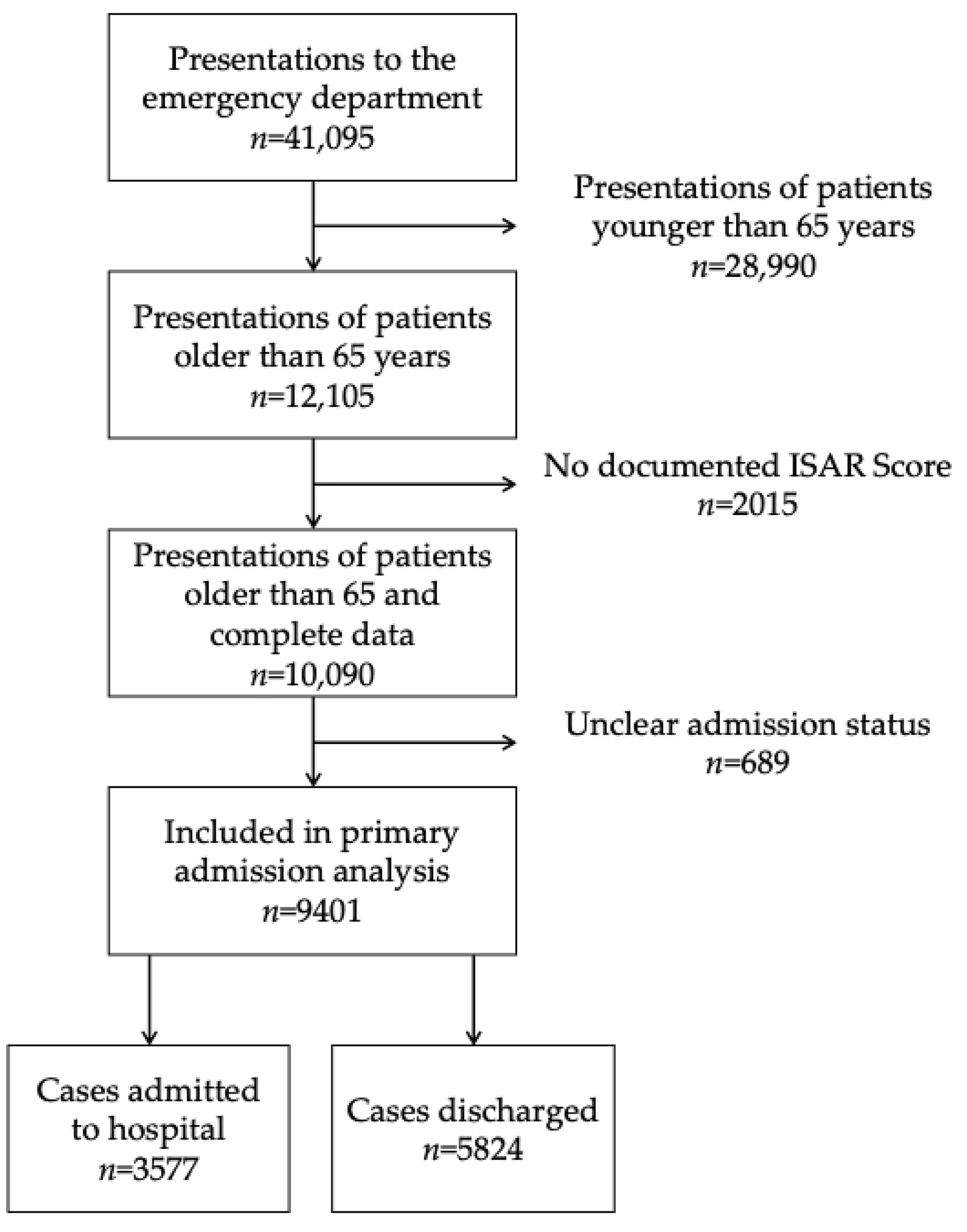
Flowchart of ED presentation inclusion and exclusion.

Compared with presentations with documented ISAR screening, presentations without an ISAR score involved slightly younger patients (median age 77 years [IQR 71–83] vs. 79 years [IQR 72–85]; *p* < 0.001), although the magnitude of this difference was small. Sex distribution did not differ significantly between groups (53.0% vs. 54.3% female; *p* = 0.257; Cramer’s V = 0.010). In contrast, triage category differed significantly (χ^2^ = 626.4, df = 4, *p* < 0.001; Cramer’s V = 0.227), with blue- and red-triaged presentations being more frequent among presentations without documented ISAR screening. The mean age of the screened population was 78.6 years (SD 8.0 years), with an age range from 65 to 105 years (Figure 1).

The distribution of ISAR scores across the 10,090 ED presentations was as follows: ISAR 0 (*n* = 2110; 20.9%), ISAR 1 (*n* = 2068; 20.5%), ISAR 2 (*n* = 2057; 20.4%), ISAR 3 (*n* = 1847; 18.3%), ISAR 4 (*n* = 1387; 13.7%), ISAR 5 (*n* = 443; 4.4%), and ISAR 6 (*n* = 178; 1.8%). Of all presentations, 54.3% (*n* = 5482) involved female patients and 45.7% (*n* = 4608) involved male patients.

Age at presentation differed significantly across ISAR categories (Kruskal–Wallis test, *p* < 0.001), with mean age increasing from 75 years for presentations with an ISAR score of 0 to 83 years for those with an ISAR score of 6. Pairwise comparisons demonstrated significant differences between most ISAR categories, whereas no significant differences were observed between ISAR scores 3 and 4, 4 and 5, 4 and 6, or 5 and 6 after Bonferroni correction.

In contrast, sex distribution remained stable across ISAR categories, with no significant differences observed between groups (χ^2^ = 3.10, df = 6, *p* = 0.796). Significant differences between ISAR categories were observed for triage acuity (χ^2^ = 447.6, df = 24, *p* < 0.001), treating specialty (χ^2^ = 66.8, df = 24, *p* < 0.001), referral source (χ^2^ = 592.7, df = 12, *p* < 0.001), and presenting complaint (χ^2^ = 198.0, df = 36, *p* < 0.001). Higher ISAR scores were associated with more high-acuity triage categories, fewer Self-presentations, and more frequent arrival via emergency medical services. Internal medicine was the most frequent treating specialty across all ISAR categories.

In the descriptive cohort, treating specialty comprised five categories, including nine presentations classified as ‘Other’; this category was not present in the final admission-analysis cohort and is therefore not displayed in Table 1 and Table 3.

### 3.2. Study Cohort for Admission Analysis

Among the 10,090 presentations with documented ISAR screening, 689 were excluded from the primary admission analysis because disposition could not be classified as hospital admission or non-admission within the predefined study framework. These comprised 535 presentations referred for external further treatment, 118 transferred directly after triage to another outpatient department within the hospital, 30 patients who died in the ED, and 6 observation cases. The final admission analysis therefore included 9401 ED presentations.

Of these, 3577 (38.0%) resulted in hospital admission and 5824 (62.0%) were classified as non-admissions. The latter comprised 5569 regular ambulatory dispositions, 204 presentations in which patients left against medical advice, and 51 presentations in which patients left before physician assessment. Baseline demographic and clinical characteristics of the study population are summarized in Table 1.

**Table 1:** Baseline characteristics of the study population stratified by hospital admission status.

Variable	Overall (*n* = 9401)	Non-Admission (*n* = 5824)	Admission (*n* = 3577)	*p*-Value
Age, years, median (IQR)	79 (73–85)	79 (73–85)	79 (72–85)	0.168
ISAR score, mean ± SD	1.99 ± 1.56	1.81 ± 1.54	2.28 ± 1.54	<0.001
ISAR score, median (IQR)	2 (1–3)	2 (0–3)	2 (1–3)	—
Sex, *n* (%)				<0.001
Female	5146 (54.7)	3334 (57.2)	1812 (50.7)	
Male	4255 (45.3)	2490 (42.8)	1765 (49.3)	
Presenting complaint, *n* (%)				<0.001
Non-specific complaints	2393 (25.5)	1333 (22.9)	1060 (29.6)	
Extremity complaints	1283 (13.6)	899 (15.4)	384 (10.7)	
Fall without head injury	1199 (12.8)	854 (14.7)	345 (9.6)	
Abdominal pain	853 (9.1)	485 (8.3)	368 (10.3)	
Head trauma	571 (6.1)	417 (7.2)	154 (4.3)	
Respiratory problems	519 (5.5)	215 (3.7)	304 (8.5)	
Other	2583 (27.5)	1621 (27.8)	962 (26.9)	
Triage category, *n* (%)				<0.001
Blue	88 (0.9)	71 (1.2)	17 (0.5)	
Green	2577 (27.4)	1993 (34.2)	584 (16.3)	
Yellow	5464 (58.1)	3339 (57.3)	2125 (59.4)	
Orange	1149 (12.2)	402 (6.9)	747 (20.9)	
Red	123 (1.3)	19 (0.3)	104 (2.9)	
Treating specialty, *n* (%)				<0.001
Trauma surgery	3779 (40.2)	2663 (45.7)	1116 (31.2)	
Internal medicine	4578 (48.7)	2579 (44.3)	1999 (55.9)	
Neurology	594 (6.3)	305 (5.2)	289 (8.1)	
General surgery	450 (4.8)	277 (4.8)	173 (4.8)	
Referral source, *n* (%)				<0.001
Self-presentation	4133 (44.0)	2899 (49.8)	1234 (34.5)	
Medical referral	991 (10.5)	493 (8.5)	498 (13.9)	
Emergency medical services	4277 (45.5)	2432 (41.8)	1845 (51.6)	

### 3.3. Comparison of Admitted and Non-Admitted ED Presentations

ED presentations resulting in hospital admission differed significantly from non-admitted presentations regarding sex, ISAR score, presenting complaint, referral source, treating specialty, and triage category (all *p* < 0.001). No significant difference in age was observed between admitted and non-admitted cases (Mann–Whitney U test, *p* = 0.168).

Presentations involving male patients resulted in hospital admission more frequently than those involving female patients (41.5% vs. 35.2%, *p* < 0.001). Likewise, higher-acuity triage categories were associated with hospital admission. Admission rates increased from 19.3% among blue-triaged cases and 22.7% among green-triaged cases to 65.0% among orange-triaged cases and 84.6% among red-triaged cases.

Admission rates also differed according to presenting complaint, with the highest rate observed among presentations for respiratory problems (58.6%). Presentations following medical referral or arrival via emergency medical services resulted in hospital admission more frequently than Self-presentations (50.3%, 43.1%, and 29.8%, respectively).

### 3.4. Association Between ISAR Score and Hospital Admission

Hospital admission rates increased progressively with increasing ISAR scores. Admission rates were 26.5% for presentations with ISAR 0, 33.9% for ISAR 1, 40.3% for ISAR 2, 45.6% for ISAR 3, 45.4% for ISAR 4, 48.3% for ISAR 5, and 48.3% for ISAR 6 (Table 2).

The most pronounced increase in admission probability was observed between ISAR scores 0 and 3, while admission rates plateaued at higher ISAR values.

Using the established screening threshold of ISAR ≥ 2, admission rates increased from 30.1% in ED presentations with ISAR < 2 to 43.9% in presentations with ISAR ≥ 2 (*p* < 0.001).

Similarly, cases with ISAR ≥ 3 demonstrated significantly higher admission rates than those with ISAR < 3 (45.9% vs. 33.4%, *p* < 0.001).

### 3.5. Logistic Regression Analysis

In the multivariable logistic regression analysis, the ISAR score remained independently associated with hospital admission. Each one-point increase in the ISAR score was associated with a 15.5% increase in the odds of admission (OR 1.155, 95% CI 1.121–1.191, *p* < 0.001). Triage category, presenting complaint, referral source, treating specialty, and sex were also independently associated with admission, whereas age was not (*p* = 0.342).

Compared with green-triaged cases, the odds of admission were higher among yellow-triaged cases (OR 1.997, 95% CI 1.781–2.239), orange-triaged ones (OR 5.057, 95% CI 4.290–5.961), and those triaged red (OR 15.071, 95% CI 9.035–25.138; all *p* < 0.001). Medical referral (OR 2.236, 95% CI 1.929–2.592) and arrival via emergency medical services (OR 1.423, 95% CI 1.282–1.580) were associated with higher odds of admission than Self-presentation. Cases assigned to neurology had higher admission odds than those assigned to trauma surgery (OR 1.546, 95% CI 1.249–1.913, *p* < 0.001), whereas no significant differences were observed for internal medicine or general surgery. The complete regression results are presented in Table 3.

In a sensitivity analysis treating ISAR as a categorical variable, model fit improved significantly compared with the model assuming a linear per-point effect (likelihood-ratio χ^2^ = 30.4, df = 5, *p* < 0.001). Compared with ISAR 0, adjusted odds of admission increased up to ISAR 3 (OR 2.020, 95% CI 1.739–2.346) and showed no further increase at higher scores, supporting a nonlinear association with a plateau at higher ISAR values.

### 3.6. Model Performance

The overall multivariable model was statistically significant (χ^2^ = 1201.2, df = 18, *p* < 0.001) and explained 16.3% of the variance according to Nagelkerke’s R^2^. At a probability threshold of 0.5, the model correctly classified 67.8% of presentations. The Hosmer–Lemeshow test was statistically significant (χ^2^ = 17.6, df = 8, *p* = 0.024).

**Figure 2:**
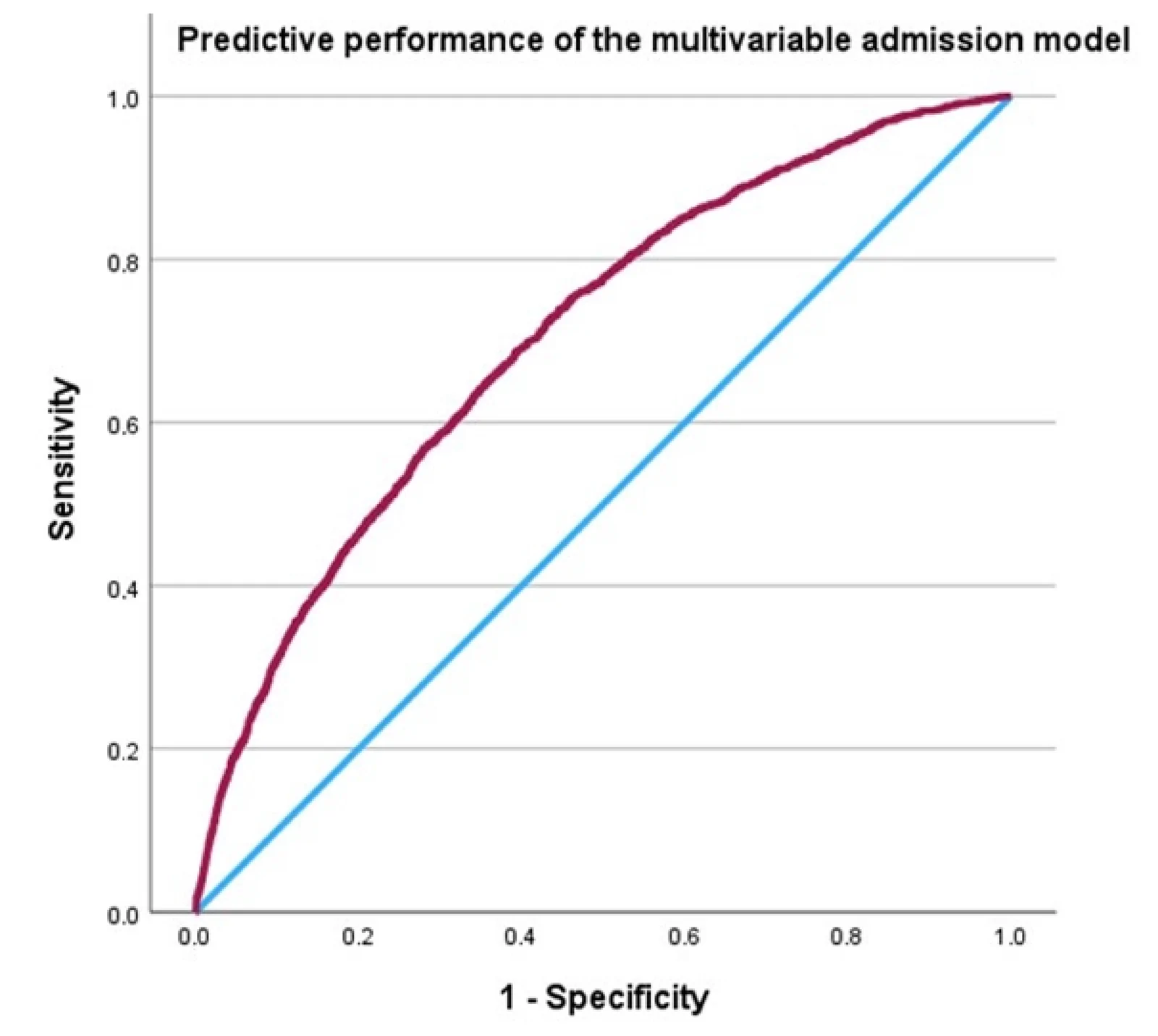
Receiver operating characteristic curve of the multivariable model for hospital admission.

The clinical model without ISAR explained 15.2% of the variance according to Nagelkerke’s R^2^. Addition of the ISAR score significantly improved model fit (Δχ^2^ = 86.0, df = 1, *p* < 0.001), increasing Nagelkerke’s R^2^ to 0.163. Discrimination improved modestly but significantly, with the AUC increasing from 0.696 for the clinical model without ISAR to 0.705 after addition of ISAR (ΔAUC = 0.009, 95% CI 0.005–0.012; *p* < 0.001). The ISAR score alone showed substantially lower discrimination (AUC 0.589, 95% CI 0.577–0.600; Figure 2).

### 3.7. Emergency Department Length of Stay

**Figure 3:**
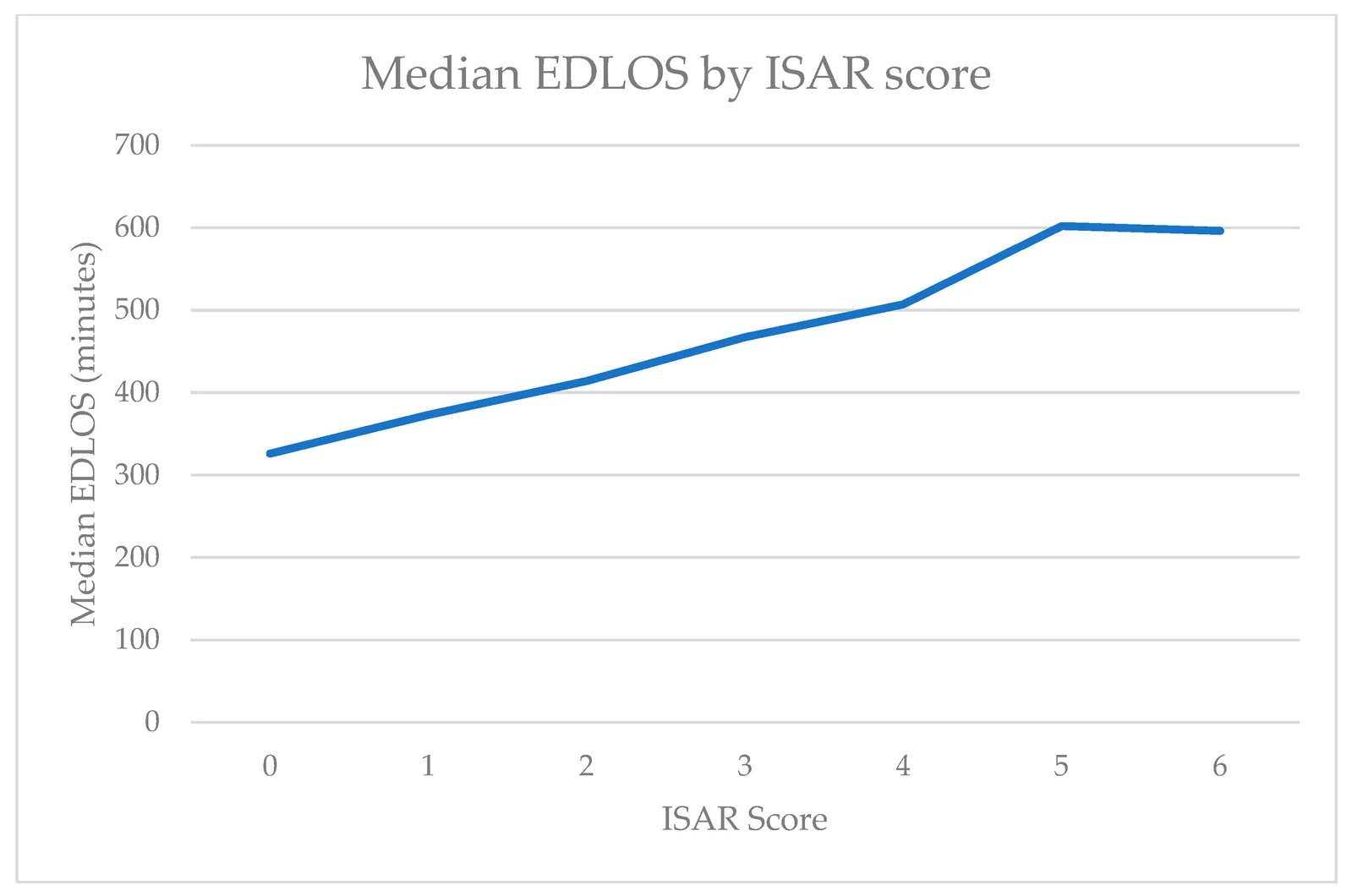
Median emergency department length of stay according to ISAR score. EDLOS increased significantly across ISAR categories (Kruskal–Wallis test, *p* < 0.001).

As a secondary outcome, emergency department length of stay (EDLOS) was analyzed according to ISAR category. The overall median EDLOS increased progressively with rising ISAR scores, ranging from 326 min in cases with an ISAR score of 0 to 596 min in cases with an ISAR score of 6. Median EDLOS increased from 326 min (IQR 215–494) for ISAR 0 to 596 min (IQR 324–1312) for ISAR 6. Median EDLOS values for ISAR scores 0 through 6 were 326 (IQR 215–494), 373 (244–584), 414 (262–698), 467 (292–968), 507 (301–1021), 602 (341–1249), and 596 (324–1312) minutes, respectively (Figure 3).

Emergency department length of stay differed significantly across ISAR categories (Kruskal–Wallis H = 530.8, df = 6, *p* < 0.001). Pairwise comparisons with Bonferroni correction revealed significant differences between most ISAR categories. Differences between the highest ISAR categories were less pronounced, with no significant differences observed between ISAR 3 and 4, ISAR 3 and 6, ISAR 4 and 6, or ISAR 5 and 6 after adjustment for multiple comparisons.

Using established ISAR thresholds, ED presentations with ISAR scores ≥ 2 demonstrated significantly longer EDLOS than presentations with ISAR scores < 2 (Mann–Whitney U test, *p* < 0.001). Similar findings were observed when applying the threshold of ISAR ≥ 3, with significantly prolonged EDLOS compared with cases scoring below 3 points (both *p* < 0.001).

To evaluate clinically relevant prolonged emergency department stays, EDLOS was dichotomized at 6 h. Overall, prolonged EDLOS occurred in 5737 ED presentations (56.9%). Among presentations with ISAR scores ≥ 2, 63.5% experienced prolonged EDLOS compared with 47.5% of cases with ISAR scores < 2 (χ^2^ = 254.0, *p* < 0.001). Likewise, prolonged EDLOS occurred in 66.3% of presentations with ISAR scores ≥ 3 compared with 51.0% among cases with ISAR scores < 3 (χ^2^ = 226.9, *p* < 0.001).

In contrast, categorical modelling of ISAR did not significantly improve model fit for prolonged EDLOS compared with the continuous specification (likelihood-ratio χ^2^ = 4.7, df = 5, *p* = 0.459), supporting the use of a linear per-point effect for this outcome.

### 3.8. Multivariable Logistic Regression Analysis for Prolonged EDLOS

Both multivariable EDLOS models included the same 9401 ED presentations with known admission status. In the initial multivariable model, the ISAR score remained independently associated with prolonged EDLOS. Each one-point increase in the ISAR score was associated with a 19.3% increase in the odds of an EDLOS ≥ 6 h (OR 1.193, 95% CI 1.158–1.230, *p* < 0.001). After hospital admission was added to the model, ISAR remained independently associated with prolonged EDLOS (OR 1.178, 95% CI 1.143–1.214, *p* < 0.001). Hospital admission itself was associated with higher odds of prolonged EDLOS (OR 1.581, 95% CI 1.437–1.739, *p* < 0.001). Age, sex, triage category, presenting complaint, referral source, and treating specialty were also independently associated with prolonged EDLOS (Table 4).

Adding hospital admission significantly improved model fit (block χ^2^ = 89.7, df = 1, *p* < 0.001), with Nagelkerke’s R^2^ increasing from 0.134 in the initial model to 0.145 in the final model. The final model correctly classified 65.0% of ED presentations and demonstrated adequate calibration according to the Hosmer–Lemeshow test (χ^2^ = 13.8, df = 8, *p* = 0.088).

**Table 4:** Multivariable logistic regression analysis for prolonged emergency department length of stay (EDLOS ≥ 6 h).

Predictor	OR	95% CI	*p*-Value
ISAR score, per point	1.178	1.143–1.214	<0.001
Age, per year	1.015	1.009–1.021	<0.001
Sex			
Female	Reference		
Male	1.104	1.011–1.204	0.027
Triage category, overall			<0.001
Green	Reference		
Yellow	1.031	0.928–1.146	0.565
Orange	0.638	0.542–0.750	<0.001
Red	0.084	0.053–0.133	<0.001
Blue	0.834	0.526–1.323	0.441
Presenting complaint, overall			<0.001
Non-specific complaints	Reference		
Extremity complaints	0.826	0.688–0.993	0.042
Fall without head injury	1.589	1.301–1.941	<0.001
Abdominal pain	1.258	1.034–1.532	0.022
Head trauma	1.805	1.420–2.293	<0.001
Respiratory problems	1.398	1.125–1.738	0.003
Other	0.777	0.685–0.882	<0.001
Referral source, overall			<0.001
Self-presentation	Reference		
Medical referral	1.013	0.875–1.172	0.864
Emergency medical services	1.722	1.557–1.905	<0.001
Treating specialty, overall			<0.001
Trauma surgery	Reference		
Internal medicine	2.153	1.866–2.484	<0.001
Neurology	1.819	1.477–2.239	<0.001
General surgery	0.805	0.614–1.055	0.115
Hospital admission			
No admission	Reference		
Admission	1.581	1.437–1.739	<0.001

## 4. Discussion

### 4.1. Principal Findings

The present study was designed to evaluate the association between the ISAR score, as a measure of geriatric risk-screening instrument, and two relevant outcomes in older emergency department patients: hospital admission and emergency department length of stay. Using a large cohort of ED presentations involving patients aged 65 years and older, we sought to investigate whether the ISAR score provides information beyond routinely available demographic and clinical parameters and whether ISAR screening may contribute to the identification of patients requiring increased healthcare resources.

Frailty has increasingly been recognized as a major determinant of outcomes among older emergency department patients. Unlike chronological age alone, frailty reflects reduced physiological reserve and increased vulnerability to acute stressors, thereby providing a more comprehensive assessment of biological age and health status. Previous studies have demonstrated associations between frailty and mortality, functional decline, institutionalization, readmission, and healthcare utilization across a variety of clinical settings [5,6,7,8,11]. The present findings extend this broader concept by demonstrating that higher ISAR scores are also associated with immediate emergency department outcomes, particularly hospital admission and prolonged EDLOS.

### 4.2. ISAR and Hospital Admission

Our findings regarding hospital admission are broadly consistent with previous investigations evaluating the ISAR score in emergency department populations. Several studies have reported increased hospitalization rates among patients with elevated ISAR scores, although the magnitude of this association has varied considerably between cohorts [18,19,20,21]. In the present study, admission rates increased from 26.5% among patients with an ISAR score of 0 to approximately 48% among patients with scores of 5 or 6.

Recent prospective data from Aksu et al. similarly demonstrated higher hospital admission rates among older ED patients with elevated ISAR scores. Their findings therefore support the association between ISAR-defined geriatric risk and immediate ED disposition observed in the present cohort [19].

Interestingly, the association between ISAR score and hospital admission was not strictly linear. Admission probability increased most prominently between ISAR scores 0 and 3 and subsequently showed a plateau at higher scores. This pattern was confirmed in a sensitivity analysis treating ISAR as a categorical rather than continuous variable, which provided a significantly better model fit. Compared with patients with an ISAR score of 0, adjusted admission odds increased progressively up to ISAR 3, whereas no further increase was observed at higher scores. These findings suggest that the incremental information provided by additional ISAR points may diminish beyond a score of approximately 3. At higher levels of vulnerability, other factors such as acute illness severity, diagnostic findings, social circumstances, or local admission practices may increasingly determine disposition decisions.

Although ISAR remained an independent predictor of hospital admission after adjustment for age, sex, triage category, presenting complaint, referral source, and treating specialty, its discriminatory performance as a stand-alone tool was modest. Each one-point increase in the ISAR score was associated with a 15.5% increase in the odds of admission (OR 1.155, 95% CI 1.121–1.191; *p* < 0.001), while the ISAR score alone achieved an AUC of 0.589. Importantly, addition of ISAR to a clinical model based on routinely available variables significantly improved model fit and increased the AUC from 0.696 to 0.705. Although this improvement was statistically significant, the absolute increase in discrimination was small (ΔAUC = 0.009), suggesting that ISAR provides incremental prognostic information but only modestly improves discrimination beyond routinely available clinical parameters. This observation is consistent with previous systematic reviews reporting only moderate predictive accuracy of ISAR across a range of outcomes [14,15,16]. Direct comparison of AUC values across studies should, however, be interpreted cautiously because previous ISAR validation studies frequently evaluated composite or longer-term adverse outcomes rather than immediate hospital admission. For example, the recently reported AUCs by Aksu et al. referred to composite adverse outcomes at 30 and 180 days and are therefore not directly comparable with the admission-specific AUC in the present study [19]. Consequently, the stand-alone AUC observed in our cohort was lower than that reported in pooled analyses but remained consistent with previous reports describing limited predictive performance of the ISAR score when used in isolation.

Importantly, however, the original purpose of the ISAR was not to function as a stand-alone decision-making instrument but rather as a rapid screening tool identifying patients at increased risk of adverse outcomes who may benefit from further geriatric assessment [17]. Consequently, the relatively limited discriminatory performance observed in our cohort should not necessarily be interpreted as a weakness of the instrument but rather as a reflection of its intended role within a broader clinical assessment strategy.

### 4.3. ISAR and Emergency Department Length of Stay

An important finding of the present study was the association between ISAR score and emergency department length of stay. Median EDLOS increased steadily from 326 min among patients with ISAR 0 to nearly 10 h among patients with ISAR 6. Furthermore, after adjustment for age, sex, presenting complaint, referral source, triage category, treating specialty, and hospital admission status, each one-point increase in ISAR score increased the odds of prolonged EDLOS by 17.8% (OR 1.178, 95% CI 1.143–1.214; *p* < 0.001).

Aksu et al. also reported longer ED stays among patients with elevated ISAR scores, providing recent prospective evidence consistent with the EDLOS gradient observed in our substantially larger real-world cohort [19].

In contrast to hospital admission, modelling ISAR categorically did not significantly improve model fit for prolonged EDLOS, suggesting that a linear per-point relationship provided an adequate approximation for this outcome. This difference may indicate that ISAR relates differently to disposition decisions and emergency department resource utilization. Hospital admission was itself independently associated with prolonged EDLOS (OR 1.581, 95% CI 1.437–1.739; *p* < 0.001), and its inclusion significantly improved model fit. Because hospital admission occurs during the same care pathway as EDLOS, its inclusion in the secondary model should be interpreted as accounting for a major disposition-related process rather than adjustment for a conventional baseline confounder.

To our knowledge, relatively few studies have specifically investigated the relationship between ISAR screening and emergency department throughput measures. Nevertheless, our findings are biologically plausible and consistent with the growing literature linking frailty to increased healthcare resource utilization [2,3,13].

Several mechanisms may contribute to the association between higher ISAR scores and prolonged EDLOS. Older patients with greater geriatric vulnerability may more frequently present with multiple concurrent medical problems, cognitive or functional impairment, or complex care needs, potentially complicating diagnostic evaluation and disposition planning. Furthermore, these patients often require multidisciplinary consultation, comprehensive discharge planning, social work involvement, or coordination of post-acute care services. Such processes may substantially increase emergency department resource utilization independent of acute illness severity.

Triage category remained independently associated with prolonged EDLOS in the fully adjusted model, although the relationship was not linear across acuity levels. This finding suggests that emergency department throughput is influenced not only by clinical urgency and geriatric vulnerability but also by differences in diagnostic pathways, treatment processes, and disposition procedures. Similarly, hospital admission status itself was associated with prolonged EDLOS, reflecting the well-recognized impact of inpatient bed availability and admission logistics on emergency department crowding.

### 4.4. Clinical Implications

From a clinical perspective, our findings suggest that ISAR screening may provide additional information on geriatric vulnerability within emergency department triage workflows. Higher ISAR scores were associated with both hospital admission and prolonged EDLOS. However, the observational design of the present study does not establish that interventions triggered by elevated ISAR scores improve patient flow, resource utilization, or clinical outcomes. ISAR should therefore be regarded as a rapid risk-screening instrument rather than a stand-alone decision-making tool.

The incremental predictive contribution of ISAR beyond routinely available clinical variables was statistically significant but modest. Addition of ISAR increased the AUC of the clinical model from 0.696 to 0.705, corresponding to an absolute increase of 0.009. Thus, ISAR may complement, but not replace, standard clinical assessment. Whether ISAR-guided strategies such as early geriatric assessment, enhanced discharge planning, or targeted resource allocation improve clinical or operational outcomes requires prospective evaluation.

### 4.5. Strengths and Limitations

The present study has several strengths. First, it includes a large cohort of more than 10,000 ED presentations involving older patients, making it one of the larger contemporary investigations of ISAR performance in routine clinical practice. Second, ISAR screening was integrated into the routine triage workflow, allowing evaluation of the instrument under real-world clinical conditions. Third, the study evaluated both a disposition-related healthcare-utilization outcome (hospital admission) and an operational emergency department outcome (EDLOS). To our knowledge, this represents one of the largest single-center cohorts evaluating routine ISAR screening in a real-world emergency department setting.

Several limitations should also be acknowledged. The retrospective single-center design limits causal inference and may affect generalizability to other healthcare systems and emergency department structures. In addition, only variables routinely available within the emergency department database could be analyzed. Although presenting complaints were included in the adjusted models, more granular diagnostic information, comorbidity burden, cognitive status, socioeconomic characteristics, availability of caregivers, laboratory findings, and comprehensive geriatric assessment results were not available for analysis. Residual confounding by unmeasured clinical and organizational factors therefore cannot be excluded. Because the dataset was pseudonymized at the encounter level and did not contain a longitudinal patient identifier, repeated ED presentations by the same individual could not be identified. Consequently, some observations may not have been statistically independent, and a sensitivity analysis restricted to the first presentation per patient could not be performed. This should be considered when interpreting the precision of the estimated associations.

ISAR documentation was unavailable for 16.6% of otherwise eligible ED presentations. Although sex distribution was similar and the difference in age was small, triage category differed between presentations with and without documented ISAR scores, with both very low- and high-acuity presentations being relatively more frequent among those without documented screening. Missing ISAR documentation therefore cannot be assumed to have occurred completely at random, and selection bias cannot be excluded.

An additional limitation is that ISAR screening was integrated into the routine triage workflow and the resulting score was available to the treating physicians. Consequently, awareness of the ISAR result may itself have influenced clinical decision-making, including hospital disposition. The observed association between ISAR and hospital admission therefore cannot be considered entirely independent of potential clinician use of the screening result. Owing to the retrospective design, the extent to which ISAR influenced individual admission decisions could not be determined.

Furthermore, several time-varying organizational factors that may influence EDLOS and hospital admission were not available in the study dataset, including time of presentation, weekday versus weekend presentation, emergency department crowding, staffing levels, and inpatient bed availability. Residual confounding by these factors cannot be excluded. EDLOS in particular should therefore be interpreted as reflecting both patient-related characteristics and organizational conditions within the emergency department and hospital. The statistically significant Hosmer–Lemeshow test for the hospital-admission model additionally indicates some deviation between observed and predicted probabilities, although this test is highly sensitive to minor discrepancies in large samples. Therefore, external validation in other emergency department settings would be desirable.

## 5. Conclusions

In this large cohort of older emergency department presentations, increasing ISAR scores were independently associated with both hospital admission and prolonged emergency department length of stay. Although the ISAR score showed only modest discrimination as a stand-alone predictor of admission, its addition to routinely available demographic and clinical variables resulted in a statistically significant but small improvement in model discrimination. These findings suggest that ISAR captures information related to geriatric vulnerability and emergency department resource utilization but do not establish that ISAR-guided interventions improve patient flow or clinical outcomes. Prospective studies are required to determine whether incorporating ISAR into targeted clinical pathways provides measurable benefit.

## Figures and Tables

**Table 2:** Distribution of hospital admission status across ISAR score categories. Admission rates increased significantly with increasing ISAR score (χ^2^ test, *p* < 0.001).

ISAR Score	Total *n*	Non-Admission *n* (%)	Admission *n* (%)
0	2037	1498 (73.5)	539 (26.5)
1	1974	1304 (66.1)	670 (33.9)
2	1916	1143 (59.7)	773 (40.3)
3	1688	919 (54.4)	769 (45.6)
4	1248	682 (54.6)	566 (45.4)
5	387	200 (51.7)	187 (48.3)
6	151	78 (51.7)	73 (48.3)
Total	9401	5824 (62.0)	3577 (38.0)

**Table 3:** Multivariable logistic regression analysis of factors associated with hospital admission.

Predictor	OR	95% CI	*p*-Value
ISAR score, per point	1.155	1.121–1.191	<0.001
Age, per year	0.997	0.991–1.003	0.342
Sex			
Female	Reference		
Male	1.203	1.100–1.316	<0.001
Triage category, overall			<0.001
Green	Reference		
Yellow	1.997	1.781–2.239	<0.001
Orange	5.057	4.290–5.961	<0.001
Red	15.071	9.035–25.138	<0.001
Blue	0.780	0.451–1.349	0.374
Presenting complaint, overall			<0.001
Non-specific complaints	Reference		
Extremity complaints	0.901	0.742–1.093	0.288
Fall without head injury	0.597	0.484–0.736	<0.001
Abdominal pain	1.367	1.127–1.658	0.001
Head trauma	0.441	0.342–0.569	<0.001
Respiratory problems	1.611	1.310–1.980	<0.001
Other	0.852	0.749–0.968	0.014
Referral source, overall			<0.001
Self-presentation	Reference		
Medical referral	2.236	1.929–2.592	<0.001
Emergency medical services	1.423	1.282–1.580	<0.001
Treating specialty, overall			<0.001
Trauma surgery	Reference		
Internal medicine	1.073	0.924–1.245	0.356
Neurology	1.546	1.249–1.913	<0.001
General surgery	0.969	0.733–1.280	0.825

## Data Availability

The datasets generated and analyzed during the current study are not publicly available due to data protection and ethical restrictions. Further inquiries may be directed to the corresponding author.

## Abbreviations

The following abbreviations are used in this manuscript:

AUC	Area under the curve
CI	Confidence interval
ED	Emergency department
EDLOS	Emergency department length of stay
EMS	Emergency medical services
IQR	Interquartile range
ISAR	Identification of Seniors at Risk
OR	Odds ratio
ROC	Receiver operating characteristic
SD	Standard deviation
